# Modulation of Nrf2 and NF-κB Signaling Pathways by Naturally Occurring Compounds in Relation to Cancer Prevention and Therapy. Are Combinations Better Than Single Compounds?

**DOI:** 10.3390/ijms22158223

**Published:** 2021-07-30

**Authors:** Violetta Krajka-Kuźniak, Wanda Baer-Dubowska

**Affiliations:** Department of Pharmaceutical Biochemistry, Poznan University of Medical Science, 4, Święcicki Street, 60-781 Poznań, Poland; vkrajka@ump.edu.pl

**Keywords:** Nrf2, NF-κB, inflammation, naturally occurring compounds, cancer chemoprevention, cancer therapy, polyphenols, phytochemical combinations

## Abstract

Nrf2 (nuclear factor erythroid 2-related factor 2) and NF-κB (nuclear factor–kappa B) signaling pathways play a central role in suppressing or inducing inflammation and angiogenesis processes. Therefore, they are involved in many steps of carcinogenesis through cooperation with multiple signaling molecules and pathways. Targeting both transcription factors simultaneously may be considered an equally important strategy for cancer chemoprevention and therapy. Several hundreds of phytochemicals, mainly edible plant and vegetable components, were shown to activate Nrf2 and mediate antioxidant response. A similar number of phytochemicals was revealed to affect NF-κB. While activation of Nrf2 and inhibition of NF-κB may protect normal cells against cancer initiation and promotion, enhanced expression and activation in cancer cells may lead to resistance to conventional chemo- or radiotherapy. Most phytochemicals, through different mechanisms, activate Nrf2, but others, such as luteolin, can act as inhibitors of both Nrf2 and NF-κB. Despite many experimental data confirming the above mechanisms currently, limited evidence exists demonstrating such activity in humans. Combinations of phytochemicals resembling that in a natural food matrix but allowing higher concentrations may improve their modulating effect on Nrf2 and NF-κB and ultimately cancer prevention and therapy. This review presents the current knowledge on the effect of selected phytochemicals and their combinations on Nrf2 and NF-κB activities in the above context.

## 1. Introduction

Dysfunction of the tumor microenvironment is related to at least 30% of cancers. Inflammatory cells, macrophages, neutrophils, or lymphocytes, the elements of the microenvironment, possess the ability to generate highly reactive species, which directly damage DNA and lead to the initiation of carcinogenesis and subsequently promote the clonal growth of the initiated cells [1]. Inflammatory cells also contribute to the process of angiogenesis. Therefore, targeting chronic inflammation and angiogenesis is considered an important strategy of both cancer chemoprevention and therapy. Nrf2 (nuclear factor erythroid 2-related factor 2) and NF-κB (nuclear factor–kappa B) signaling pathways, activated by various stimuli, play a central role in suppressing or inducing inflammation and angiogenesis processes.

Moreover, although Nrf2 and NF-κB transcription factors are directly involved in many steps of carcinogenesis, cooperation with multiple other signaling molecules and pathways may ultimately affect cell differentiation and proliferation [2]. Since interference exists between these two pathways, their concomitant modulation, i.e., induction of Nrf2 and inhibition of NF-κB in normal cells and inhibition of both in cancer cells, may be considered the best strategy of cancer chemoprevention and therapy, respectively. Modulation of multiple signaling pathways is an element of the therapeutic approach named anakoinosis [3]. The concept of anakoinosis is an alternative to conventional chemotherapy, which is usually based on a single target or focused on a single area of the tumor and can equally be applied in chemoprevention.

Several hundreds of phytochemicals, mainly edible vegetables and fruits components, were shown to activate Nrf2 and mediate antioxidant response. One of the first in this group was naturally occurring and subsequently chemically modified triterpenoids [4]. Similar numbers of phytochemicals were shown to modulate the NF-κB pathway. In this group, curcumin (diferuloylmethane) was one of the first widely described as an inhibitor of NF-κB in cancer cells [5]. Among them, only a relatively small amount affected both pathways, and several of their modulators reached clinical trials level [6].

The activity of these phytochemicals was assessed mainly as single compounds but also in the natural food matrix. Recently, attempts were made to select the best combination of naturally occurring compounds for chemoprevention or therapeutic purposes, including modulation of Nrf2 and NF-κB pathways.

This review focuses on presenting the comparison of the modulating effect of selected phytochemicals and their combinations on Nrf2 and NF-κB activities in relation to their possible chemopreventive or therapeutic applications.

## 2. Overview of Nrf2 and NF-κB Signaling Pathways and Their Interconnections

The Nrf2 pathway is responsible for cytoprotection. Its activation leads to the expression of several cytoprotective enzymes and proteins involved in cellular defense against reactive oxygen species (ROS) and electrophilic species [7].

Therefore, it represents the first line of defense against cancer initiating and promoting agents. Under normal conditions, Nrf2 is sequestered in the cytoplasm bound to its repressor Keap1 (Kelch-like ECH associated protein 1). Keap1 is rich in cysteine residues protein and is divided into five domains [8]. Polyubiquitination by Keap1/Cul3 ubiquitin ligase leads to rapid Nrf2 degradation by proteasomes [9].

Nrf2 activators, including phytochemicals, such as naturally occurring isothiocyanates, bind to SH groups of the Keap1 protein, leading to a conformational change precluding Nrf2 degradation and allowing its translocation into the nucleus. In the nucleus, Nrf2 heterodimerizes with small Maf proteins (MafF, MafG, and MafK) through its bZip domains and binds to the ARE 5′-GTCACAGTGACTCAGCAGAATCTG-3′ target sequence in DNA [10].

Besides the canonical pathway of activation, Nrf2 can also be regulated by phosphorylation. Post-translational modification of Nrf2 by various protein kinases can affect the release of Nrf2 from the complex with Keap1, its nuclear translocation, and stability [9]. In one of the mechanisms of the non-canonical pathway of Nrf2 activation, the p62 (SQSTM1) protein is involved. This protein is an autophagy receptor for protein and mitochondria degradation. Similar to the interaction of Keap1 with Nrf2, p62 protein is able to interact with Keap1 by the KIR domain in p62. The interaction of Keap1 with p62 induces a dependent autophagy degradation of Keap1 and subsequent Nrf2 stabilization and activation in MEF and HEK293 cells [11].

The Nrf2 activation dependent on p62 increases the expression of NAD(P)H:quinone oxidoreductase (NQO1), glutathione -S-transferases (GSTs) and anti-apoptotic proteins such as Bcl-2 (B-cell lymphoma 2) and Bcl-xL (B-cell lymphoma- extra-large), decreasing ROS levels and protecting the cell against oxidative stress [10]. However, sustained Nrf2 activation by impairment of autophagy and an increase in p62 phosphorylation promotes cancer cell proliferation. Mutations in the KIR domain in p62, which prevents Keap1-p62 interaction, is associated with a ROS increase [10,12]. Epigenetic mechanisms, such as Nrf2 or Keap1 promoter methylation and microRNA have also been implicated in the complex regulation of Nrf2 pathway activity [9].

Although Nrf2 pathway plays an essential role in maintaining cellular redox and electrophilic homeostasis, it was also demonstrated that Nrf2 is overexpressed in cancer cells and may contribute to increased proliferation, invasion, and chemoresistance [13]. Several mechanisms are involved in the prooncogenic activation of the Nrf2 pathway in cancer cells and include both genetic alterations and epigenetic changes [14]. Evidence exists that this pathway is associated with the proliferation of cancer cells through metabolic reprogramming [15].

Thus, induction of Nrf2 activation by phytochemicals in normal cells or at the early stages of carcinogenesis is an important strategy of chemoprevention. In cancer cells, the naturally occurring inhibitors of this pathway are desired.

While Nrf2 protects against reactive species and, in general, inflammatory insult, NF-κB plays a crucial role in inflammatory response and production of pro-inflammatory molecules, including cytokines and enzymes such as cyclooxygenase-2 (COX-2) and inducible nitric oxide synthetase (iNOS). These enzymes are involved in the promotion of carcinogenesis as well as angiogenesis. NF-κB is found in immune cells such as neutrophils and is an important anti-apoptotic transcription factor. Moreover, it is essential for the development of lymphocytes [16].

The NF-κB protein family is composed of five structurally related members, including NF-κB1 (also named p50), NF-κB2 (also named p52), RelA (also named p65), RelB, and c-Rel, which mediates transcription of *COX-2* and *iNOS*, its major target genes, by binding to a specific DNA element, κB enhancer, as various hetero- or homo-dimers [17]. Dimer p50/p65 is the most common active form of NF-κB. In this complex, the p65 subunit is responsible for initiating transcription, while the p50 subunit serves only as a helper in NF-κB DNA binding [17,18]. In a normal state, the NF-κB subunits are sequestered in the cytoplasm by inhibitory proteins, mainly IκB family members, and similarly as Nrf2, which can be activated by canonical and non-canonical mechanisms [12].

In the canonical pathway, activation of the IκB kinase (IKK) complex leads to phosphorylation and ubiquitination of IκB and subsequent degradation by 26 s proteasome. The released active dimers migrate into the nucleus and activate the transcription of the target genes [19].

The noncanonical NF-κB activation does not involve IκBα degradation, but instead relies on the processing of the NF-κB-precursor protein, p100, and is a selective response to a specific group of stimuli, including ligands of a subset tumor necrosis factor receptor (TNFR) superfamily members such as LTβR, BAFFR, CD40, and RANK [12].

In the nucleus, the active NF-κB complex binds to its target of the 5′-GGGRNYY YCC-3′ sequence [20] and connects with the basal transcriptional machinery. Its association among the others with AP-1 (c-Jun/c-Fos complex) and chromatin remodeling proteins, such as CREB-binding protein (CBP) and p300 is also possible [21].

Several factors influencing NF-κB activation and nuclear translocation may be responsible for the diversity of NF-κB and sometimes opposing roles as a pro- and anti-inflammatory mediator [16]. In this regard, NF-κB is well-known for providing cancer cells with a survival advantage by upregulating anti-apoptotic genes. Moreover, there is reciprocal cross-talk between NF-κB and autophagy in cancer. Therefore, NF-κB can either promote or repress tumorigenesis, depending on the stimulus and the context [22].

**Figure 1:**
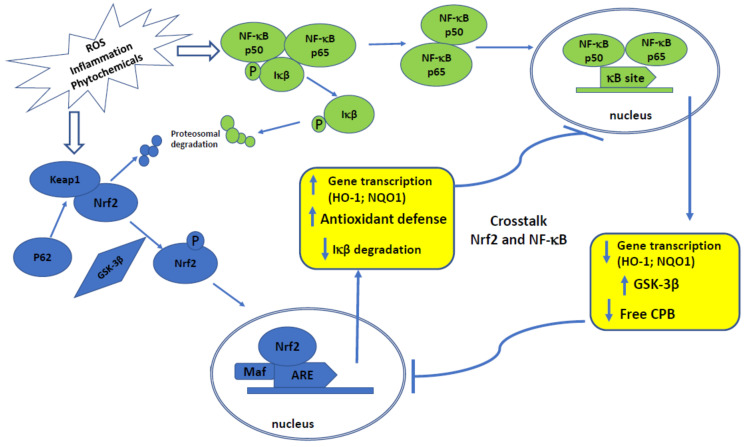
Shematic presentation of crosstalk between Nrf2 and NF-κB signaling pathways and their activation.

Importantly, downregulation of NF-κB negatively interferes with the Nrf2 signaling pathway (Figure 1).

In this regard, Nrf2 reduces reactive oxygen species (ROS) levels and prevents IĸB-α proteasomal degradation and arrests nuclear translocation of NF-ĸB. Furthermore, up-regulation of Nrf2 enhances heme oxygenase (HO-1) levels, and a subsequent enhancement in phase II enzymes expression blocks the degradation of IĸB-α [8].

Several pieces of evidence indicated that Nrf2 opposes the NF-κB by clashing with the transcription co-activator cAMP response element (CREB) binding protein (CBP) [23]. The CBP-p300 complex is responsible for the acetylation of histones and of non-histone proteins, including Nrf2 and p65 [24]. CBP connects with Neh4 and Neh5 domains of Nrf2, leading to the acetylation of the Neh1 domain, which is responsible for DNA-binding. In addition, CBP interacts with phosphorylated p65 at Ser276, thus in the case with the overexpression of p65, it inhibits the availability of CBP for Nrf2 connection [25].

Moreover, genes such as encoding *NQO1*, *glutamate-cysteine ligase* catalytic subunit, and *glutamate-cysteine ligase* modifier subunit, whose expression is controlled by Nrf2, also possess NF-κB binding site. This relationship indicates that Nrf2 and NF-κB signaling pathways may cooperate in promoting chemoresistance of cancer cells in response to drugs treatment [8].

An interesting link between Nrf2 and NF-κB presents glycogen synthase kinase-3β (GSK-3β). GSK-3β can phosphorylate Nrf2 on tyrosine 568, resulting in activation of this factor. Furthermore, the same enzyme has been reported to be necessary for the full transcriptional activity of NF-κB, demonstrating that GSK-3β selectively supports the expression of a subset of genes activated by NF-κB-dependent proliferative signals [26].

Therefore, targeting both signaling pathways by naturally occurring compounds is considered an efficient strategy of both chemoprevention and supporting conventional therapy. However, “double faces” of both Nrf2 and NF-κB must be considered.

## 3. Phytochemicals as Modulators of Nrf2 and NF-kB Signaling Pathway

The term phytochemicals is usually used to describe chemicals from plants that may affect health but are not essential nutrients. Considering their chemical structure, the major representative classes of these compounds are polyphenols, organosulfur derivatives, terpenoids, and phytosterols.

Polyphenols are a diversified group of phytochemicals and include five major subclasses, simple phenols and phenolic acids, hydroxycinnamic acid derivatives, stilbene derivatives, flavonoids, and polymeric polyphenols such as procyanidins derived from proanthocyanidins, also named condensed tannins [27,28].

This is the most extensively studied group of phytochemicals, as it illustrates the growing number of publications, reaching a number of more than 20,000 publications from 2000–2016 [29].

**Figure 2:**
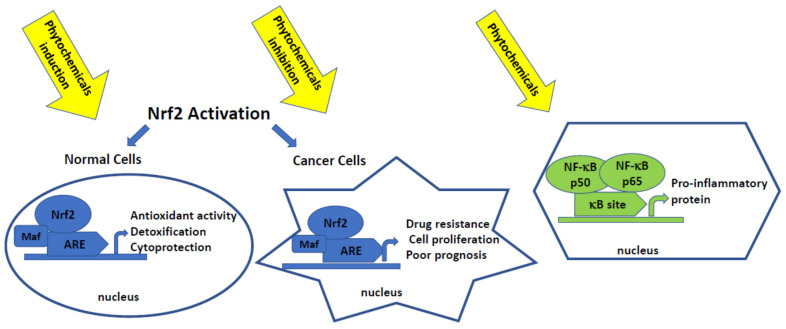
Shematic presentation of the activation of Nrf2 and NF-κB pathways. Phytochemicals as single compounds or their combinations affecting these pathways may act in the early stages of carcinogenesis as chemopreventive agents or support convention therapy protecting against chemoresistance.

Accumulating evidence indicates that most phytochemicals can target a wide variety of signaling pathways and molecules ultimately implicated in cellular differentiation, proliferation, and cell death [30]. Moreover, these effects are often related to the modification of Nrf2 and/or NF-κB pathways (Figure 2) [31].

### 3.1. Phytochemicals and Nrf2-Handling Its Double Faces toward Cancer

The Nrf2 pathway plays mainly a cytoprotective role. Therefore, the effect of phytochemicals on its activation and subsequently the expression of its target genes was the subject of several studies in the context of prophylaxis of many diseases, particularly with inflammation background, thus including cancer.

In this regard, activation of Nrf2 was used to measure the outcome of phytochemical effectiveness in human intervention trials. The recent review by Clifford et al. [32] provided a systematic summary of the evidence for the administration of dietary phytochemicals to induce Nrf2 in humans. As single components, two representatives of polyphenols class of phytochemicals resveratrol and curcumin, sulforaphane–organosulfur derivative and lycopene-carotenoid were assessed, and total Nrf2 DNA binding level or total Nrf2 gene expression were evaluated. They concluded that there is insufficient high-quality evidence indicating that phytochemicals activate Nrf2 in humans. Therefore, it is still critical to examine if the positive findings reported in cell culture and animal studies are translatable to humans.

In vitro or in vivo in animal models, the most extensively studied polyphenols were tea components, particularly epigallocatechin-3-gallate and epicatechin, resveratrol, and curcumin, along with several other components of fruits, vegetables, and spices.

The results of these studies were described in many comprehensive reviews in the context of prevention of different diseases, including cancer [27,29,33].

The less explored are specific polymeric polyphenols such as tannins, betalains, pigments derived from betalamic acid, and prenylflavonoids such as xanthohumol. However, these naturally occurring compounds are also potent modulators of signaling pathways including Nr2.

In this regard, tannic acid, ester of gallic acid, and glucose was shown to modulate phase II enzymes which expression is regulated by Nrf2, both in animal models and in cell cultures in vitro, leading to deactivation of ultimate metabolites of carcinogens. In animal models, this effect was, to some extent, tissue specific. In cell cultures the anti-or prooxidant activity related to Nrf2 modulation depended on tannic acid concentrations [34].

Betalains are components of beetroot and have been reported to have high antioxidant and anti-inflammatory activity in vitro and in vivo in a variety of animal models [35]. The most extensively studied representative of these compounds is betanin.

The “direct” antioxidant power of betanin, which removes reactive oxygen or nitrogen species, is related to the hydroxyl group of the phenol residue and the cyclic amine group, similar to the phenolic antioxidant ethoxyquin, which are good hydrogen donors and confers reducing properties to this class of compounds [36].

The influence of betanin on the activation of Nrf2 and the expression of GST isozymes GSTA, GSTP, GSTM, GSTT, NQO1, and HO-1 was assessed in hepatic non-tumor THLE-2 and hepatoma-derived HepG2 cell lines. The results of this study indicated that betanin may exert hepatoprotective and anticarcinogenic effects through the activation of Nrf2 and subsequent induction of the expression of genes controlled by this factor. In addition, the activation of mitogen-activated protein kinases may be responsible for the activation of Nrf2 in the THLE-2 cells [37].

Xanthohumol is a prenylated flavonoid that is found within the inflorescence of *Humulus lupulus L*. (hop plant). Therefore, it is an important ingredient in beer. Several studies evaluating the anti-cancer potential of xanthohumol showed its effectiveness in different cancer models in vitro and in vivo [38], partly via induction of the Nrf2 pathway. In this regard, xanthohumol increased the expression and led to the activation of Nrf2 in immortalized normal THLE-2 hepatocytes and a hepatocellular carcinoma HepG2 cell line. However, in contrast to normal cells, the expression of genes controlled by this transcription factor was not affected in HepG2 cells, except for GSTA and GSTP. Including Besides the induction of GSTs and HO-1, Xanthohumol significantly elevated NQO1 expression in concert with p53 levels in normal hepatocytes. Therefore, the activation of the Nrf2 pathway, and subsequently, phase II enzymes in concert with p53 induction in normal hepatocytes may account for the molecular mechanism of the chemopreventive activity of xanthohumol [39].

Xanthohumol was also shown to exert anti-inflammatory activity through Nrf2 signaling and up-regulation of downstream HO-1 in mouse microglial BV2 cells, suggesting its role in regulating inflammatory responses in the brain [40]. Xanthohumol, via induction of AMPK/GSK3β-Nrf2, signals axis-ameliorated lipopolysaccharide (LPS)-induced acute lung injury [41].

The differences in the effect of xanthohumol and the other hop-derived prenylflavonoids in proliferating and differentiated colorectal cancer cells (CaCo-2 cells) were demonstrated. In the latter, expression of phase II enzymes also showed specificity toward their isozymes, namely GST [42].

Ursolic acid is a natural triterpenoid found in abundance in blueberries, cranberries, and apple peels. In mouse epidermal JB6P+ cells, this triterpenoid induced the expression of the Nrf2-mediated detoxifying and antioxidant enzymes HO-1, NQO1, and UDP-glucuronosyltransferase 1A1. Furthermore, DNA methylation analysis and expression of the epigenetic enzymes DNA methyltransferases and histone deacetylase revealed that epigenetic effects may be responsible for Nrf2 induction and activation and potential beneficial effects of ursolic acid, including the prevention of skin cancer [43].

The above examples refer to activation of Nrf2 activation by phytochemicals. This is an important chemoprevention strategy.

However, often occurring in cancer cells, overexpression of Nrf2 due to both genetic and epigenetic mechanisms prompt the searching of naturally occurring compounds that inhibit its activity and expression to avoid chemo -or radiotherapy resistance.

Among the naturally occurring compounds, flavonoids, luteolin, and apigenin decreased Nrf2 mRNA and protein levels in human non-small cell lung cancer NSCLC/A549 cells and hepatocellular carcinoma HCC/Bel-740ADM cells [44,45]. Similarly, procyanidins prepared from *Cinnamomi* cortex extract suppressed Nrf2 activity and expression in human A549 NSCLC cells [46]. Certain Nrf2 inhibitors have been recommended for the treatment of Nrf2-overexpressed cancers. Among them, the most widely described Nrf2 inhibitor is a natural quassinoid brusatol, which stimulates poly-ubiquitination of Nrf2 and decreases the Nrf2 protein level [14].

Recently, a mechanism of Nrf2 inhibition leading to cell death was described for quercetin, the most extensively studied flavonoid. The experiments were performed in vivo in human xenograft acute myeloid leukemia (AML) models and in vitro using leukemia cell lines and showed that quercetin may induce apoptosis in part by decreasing Nrf2 nuclear translocation, inducing Nrf2 proteasomal degradation and downregulation of histone deacetylase HDAC4, which leads to up-regulation of pro-apoptotic miRNAs.

A previous study of the same group showed that quercetin-induced apoptosis, partly due to its DNA demethylating activity, through inhibition of HDAC and modification of histone H3ac and H4ac, leads to transcription of genes whose products are involved in the apoptosis pathway [47].

Therefore, epigenetic regulation of Nrf2 expression and activity may be considered an important target of phytochemicals acting as inducers or inhibitors of this pathway. Phytochemicals, such as ursolic acid and quercetin along with curcumin, resveratrol, and sulforaphane, paved the way to explore this mechanism of Nrf2 modification [48].

In summary, naturally occurring compounds through different mechanisms influence the Nrf2 pathway and ultimately may contribute to cancer prevention or support cancer therapy.

### 3.2. Phytochemicals and NF-κB–Inhibitors Are Needed in Cancer Chemoprevention and Therapy

Although NF-κB is essential for the function of both pro-inflammatory and regulatory immune cells and can have a dichotomic role on tumoral immunity, depending on the type of immune cells present in the cancer microenvironment, inhibiting NF-κB activation is an important strategy of both chemoprevention and chemotherapy [16].

Several phytochemicals, which showed the ability to activate Nrf2 pathway, also inhibit NF-κB activation.

In this regard, polyphenols representative of all aforementioned subclasses, such as epigallocatechin gallate, resveratrol, curcumin, luteolin and quercetin as well as the organosulfur compound diallyl disulfide, were shown to act along with other mechanism through inhibition of NF-κB activation [27,31]. These compounds were also clinically tested [6]. Low bioavailability was indicated as their major limitations.

Curcumin, one of the most extensively studied phytochemicals and one of the first described inhibitors of NF-κB [49] is an potent anti-inflammatory agent able to protect against different types of cancers in vitro and in vivo [6].

However, none of double blinded, placebo controlled clinical trials of curcumin have been successful. The reason may be low bioavailability and stability along with high reactivity of this phytochemical [50].

Among the less studied polyphenols, tannic acid was demonstrated to inhibit NF-κB activation and subsequent expression and activity of iNOS and COX-2 enzymes, the key players of the inflammation process in mouse epidermis stimulated by 12-*O*-tetradecanoyphorbol-13-acetate [51]. Betanin and other betalains were shown to be potent inhibitors of COX-2 expression and activity indirectly indicating the modification of NF-κB pathway [52]. The effect of xanthohumol on NF-κB activation was shown in pancreatic cancer cells (PANC-1). This prenylated flavonoid inhibited the NF-κB active complex binding to DNA and subsequent expression of *COX-2* gene [53].

Ursolic acid is described as one of the first inhibitors of NF-κB. Recently, microarray and CMAP analysis in breast cancer MCF-7 cells showed that ursolic acid may exert anti-tumor activity by inhibiting IKK/NF-κB along with the RAF/ERK pathway. Reduced phosphorylation of PLK1 may be involved in this effect [54].

Many other phytochemicals showing anti-inflammatory effects by inhibiting NF-κB signaling also affect the MAPK signaling pathway. These include curcumin, flavonoid mangiferin, and sesquiterpene lactone parthenolide [31].

There are many data on the effect of phytochemicals on Nrf2 or NF-κB pathways. However, relatively less data refer to the influence of the given naturally occurring compounds on both of these pathways in the same model simultaneously. Examples are presented in Table 1.

### 3.3. The Effect of Food Matrix-A Prototype of Combinations Strategy on Nrf2 and NF-κB Activities

Most phytochemicals with chemopreventive or therapeutic potential derive from edible fruits and vegetables. Therefore, the influence of a natural food matrix on Nrf2 and NF-κB signaling pathways was the subject of several studies, including human intervention trials. The tested products were administered as juices, extracts, or in forms used in a regular diet, such as virgin olive oil.

In this regard, in the review summarizing the clinical trials in which Nrf2 was used as a measure of outcome [32], the results of administration of normalized coffee extract, bilberry pomace extract, virgin olive oil, seed (sunflower) oils, cherry juice, grape, and broccoli sprout extracts treatment are described. The increase of total Nrf2 gene expression was observed and this effect was dependent on duration of trial and time point. In general, higher risk bias was noticed in comparison with single compounds. A relatively less explored food matrix is beetroot (*Beta vulgaris* L.), containing several bioactive compounds, including unique betalains.

Early studies on beetroot juice on DMBA treated rats showed increased activity of phase II enzymes in the liver and mammary gland, indirectly indicating the induction and activation of Nrf2 [77]. However, beetroot water extracts were not found to induce phase II enzymes in human colon adenocarcinoma cells in vitro [78]. The protective effect of beetroot juice against rat liver injury and inflammation induced by carcinogenic N-nitrosodiethylamine (NDEA) was also demonstrated. In comparison with the untreated group of animals, the beetroot juice provided significant defense against a range of inflammatory markers induced by NDEA administration in rat liver [79]. Increased level and activity of NQO1 was the most significant change among phase II enzymes. Beetroot juice reduced the DNA damage increased as the result of NDEA treatment, and the biomarkers of liver injury. These observations, again indirectly, indicated modulation of Nrf2 and NF-κB by beetroot juice. In another study, the protective effect of beetroot extract on gentamicin-induced nephrotoxicity in rats through downregulation of NF-κB and the nitric oxide level was observed [80].

Modulation of phase II enzymes in rats was also observed as result of treatment with cloudy apple juice rich in procyanidins and other polyphenols. The latter reduced inflammation response of the kidneys in unilateral ureteral obstruction rats via decreasing the expression and activity of COX-2, downregulating the transcription factor NK-κB and up-regulating the expression of Nrf2 [81].

The above examples indicate that, in different ways, several fruits, vegetables, and herbs preparations affect cellular signaling pathways involved in cancer development, including Nrf2 and NK-κB.

Implementation of natural food rich in phytochemicals activating Nrf2 and reducing the activity of NK-κB is the best way of cancer chemoprevention. However, the low concentrations of active compounds in the natural food matrix limits the effectiveness of this strategy.

### 3.4. Phytochemical Combinations Affecting Nrf2 and NF-κB Signaling Pathways

In general, the low toxicity and capability of inhibiting or inducing multiple signaling pathways, including Nrf2 and NF-κB, represent a resourceful long-term strategy for chemoprevention or treatment of cancer. It may be advantageous to limit compensatory signaling feedback loops, crosstalk existing among cellular pathways, and different cell types inside the tumor microenvironment. The broad molecular diversity offered by natural compounds prompts the identification of synergistic combinatorial treatments [82].

The results of several studies in vitro, and to a lesser extent, in animal models indicate that combinations of phytochemicals may increase their chemopreventive and chemotherapeutic potential and can efficiently target the signaling pathways involved in cell proliferation and survival.

In this regard, it was demonstrated that a combination of ursolic acid and curcumin more efficiently inhibited the promotion of mouse skin tumors through downregulation of key inflammatory elements, including NF-κB [83], compared to the single compounds. Similarly, a combination of quercetin, kaempferol, and pterostilbene synergistically attenuated ROS through the activation of the Nrf2 signaling pathway in hepatic cells. Specifically, treatment with this combination significantly induced Nrf2 binding to ARE sequence and increased the mRNA and protein expression of Nrf2-regulated genes in human hepatoma cells HepG2–8. Therefore, this study demonstrated that the berry constituents, quercetin, kaempferol, and pterostilbene, activate the Nrf2 signaling pathway and exhibit synergistic anti-oxidative stress activity at appropriate concentrations [84].

The effect of the combination of phenethyl isothiocyanate, indole-3-carbinol, xanthohumol, and resveratrol on the expression and activation of NF-κB was evaluated in pancreatic cancer cells. The mixture of xanthohumol and phenethyl isothiocyanate was more efficient than the single compounds in reducing the NF-κB activation by diminishing binding of NF-κB p65 subunit to DNA and expression of the *p65* gene. The same combination also enhanced activation and expression of Nrf2 and ultimately its target genes *GSTP*, *NQO1*, and superoxide dismutase gene *SOD*. Modulation of these two pathways resulted in reduced proliferation of pancreatic cancer PANC-1 cells. In addition, increased Yeslevels of P-Nrf2 and P-JNK and a decreased level of P-GSK-3β suggested these kinases are involved in the activation of Nrf2. Moreover, the mixture of xanthohumol and phenethyl isothiocyanate induced cell cycle arrest (G0/G1 phase) and increased apoptosis and autophagy markers [53]. These findings indicate that combinations of phytochemicals resembling that which occurs in natural diets may efficiently modulate the Nrf2 and NF-κB signaling pathways and limit pancreatic cancer cell survival and proliferation. Therefore, it may be applicable in cancer prophylaxis or in improving the results of conventional therapy.

Table 2 shows more examples of phytochemicals combinations that act on both Nrf2 and NF-κB and are more potent modulators of these pathways than single compounds.

In most of the in vitro studies, equimolar concentrations of the tested phytochemicals were applied. Only in several cases did arbitrary selection occur. The in vivo studies in the context of this review are sparse thus far. Therefore, the examples shown in Table 2 refer mostly to the inflammation model. Concerning the mode of interaction between the phytochemicals, only synergetic effects were observed. However, evaluation based on the Chou–Talalay method of the nature of interaction was rarely employed.While the combinations of phytochemicals may be more efficient, particularly in chemoprevention, naturally occurring compounds may have the potentially inhibitory and antagonistic effect on chemotherapeutics’ activity when used in support of conventional therapy. Therefore, the identification of phytochemicals acting in this way can potentially explain causes for drug failure or resistance and propose the timing and use of naturally occurring compounds along with cancer chemotherapy [85]. In the context of Nrf2 interference with cancer chemotherapy, its status of anti- or pro-tumorigenic is defined by many different modalities, but mainly the loss of functional Keap1 or its mutation contribute to deregulation of Nrf2 in cancer cells. However, it was demonstrated using synthetic triterpenoid RTA 405 that pharmacological activation of Nrf2 may be distinct from genetic activation and does not provide a growth or survival advantage to certain cancer cells, including pancreatic cancer cells. Moreover, pre-treatment with RTA 405 did not protect cancer cells from doxorubicin- or cisplatin-mediated growth inhibition [86]. However, considering the complex mechanism of Nrf2 overexpression in cancer cells, the increased activation of this transcription factor by phytochemicals should be avoided as it may enhance chemoresistance.

**Table 2:** Modulation of Nrf2 and NF-κB pathways by selected combination phytochemicals in vitro and in vivo model.

Phytochemicals Combination	Phytochemicals Interactions	Experimental Model	Concentrations	Effect on NF-ĸB	Effect on Nrf2	Ref.
Resveratrol and Phenethyl isothiocyanate	Synergism	Human Pancreatic cancer cells (Mia-Pa-Ca-2 cells)	* Resveratrol 10 µM; Phenethyl isothiocyanate 10 µM		↑ expression of Nrf2 and binding Nrf2 to DNA, and expression of SOD, NQO1, GSTP	[87]
	Synergism	PANC-1 cells	Resveratrol 10 µM; Phenethyl isothiocyanate 10 µM	↓ binding NF-ĸBp65 to DNA and expression of NF-ĸBp65 and COX-2		[53]
Xanthohumol and Phenethyl isothiocyanate	Synergism	PANC-1 cells	Xanthohumol 10 µM; Phenethyl isothiocyanate 10 µM		↑nuclear translocation of Nrf2, and binding Nrf2 to DNA, and expression of Nrf2, SOD, NQO1, GSTP	[53]
	Synergism	PANC-1 cells	Xanthohumol 10 µM; Phenethyl isothiocyanate 10 µM	↓ nuclear translocation NF-ĸB, and binding NF-ĸBp65 and NF-ĸBp50 to DNA, and expression of NF-ĸB and COX-2		[53]
Curcumin and Arctigenin	Synergism	Human prostate adenocarcinoma cells (LNCaP cells); MCF-7 cells	Curcumin 5 μM, Arctigenin 1μM	↓ phosphorylation of NF-ĸB; and p-IκB levels		[88]
Curcumin and Epigallocatechin gallate	Synergism	LNCaPcells; MCF-7 cells	Curcumin 5 μM; EGCG 40 μM	↓ phosphorylation NF-ĸB; ↓ p-IκB levels		[88]
3,3′-Diindolylmethane and Sulforaphane	Additive	Human liver hepatoma cells (HepG2-C8 cells)	3,3′-diindolylmethane 6.25µM; Sulforaphane 1µM		↑ expression of Nrf2 and SOD	[89]
Sulforaphane and Curcumin	Synergism	RAW264.7 cells	Sulforaphane 0.4 μM; Curcumin 2 μM	↓ expression of iNOS; COX-2; PGE2		[90]
	Synergism	RAW264.7 cells	Sulforaphane 0.4 μM; Curcumin 2 μM		↑ expression of Nrf2 and NQO1, HO-1	[90]
Sulforaphane and Phenethyl isothiocyanate	Synergism	RAW264.7 cells	Sulforaphane 0.4 μM; Phenethyl isothiocyanate 2 μM	↓ expression of iNOS; COX-2; PGE2		[90]
	Synergism	RAW264.7 cells	Sulforaphane 0.4 μM; Phenethyl isothiocyanate 2 μM		↑ expression of Nrf2 and NQO1, HO-1	[90]
Curcumin and Resveratrol	Synergism	Human hypopharyngeal carcinoma cells (Fadu cells) Human oral adenosquamous carcinoma cells (Cal-27 cells)	Curcumin 25 μM; Resveratrol 25 μM	↓ nuclear translocation of NF-ĸB		[91]
	Synergism	Xenografts SCID mouse Spinal cord injury model	** Curcumin 500 mg/kg; Resveratrol 150 mg/kg gavage	↓ NF-ĸB binding to DNA		[92]
Curcumin and Piperine	Lack of Synergism	Holtzman rats Periodontitis model	Curcumin 400 mg/kg; Piperine 20 mg/kg gavage	↓ phosphorylation and activation of NF-ĸB		[93]

* Concentrations of phytochemicals in in vitro studies are quoted in μM; ** Concentrations of phytochemicals in vivo studies are quoted in mg/kg.

## 4. Conclusions and Perspectives

The existing cross-talk between Nrf2 and NF-κB key inflammation players may be an effective target of bioactive naturally occurring compounds. Moreover, these pathways are important elements of the signaling network ultimately involved in the regulation of cell proliferation and death.

As Nrf2 activation in normal cells or at early stages of carcinogenesis may protect against DNA insult and angiogenesis through the enhanced expression of 250 genes under the control of Nrf2, and protects against cancer development in cancer cells may contribute to resistance to chemotherapy.

Therefore, for cancer prophylaxis, naturally occurring activators or inducers of the Nrf2 pathway and inhibitors of NF-κB are required. In contrast, for cancer treatment, inhibitors of both of these pathways are investigated.

While many phytochemicals acting either as inducers or inhibitors of Nrf2 pathways and inhibitors of NF-κB were described, relatively less was shown to act simultaneously in the same experimental model. Moreover, the evaluation of a modulating effect of phytochemicals mainly addressed the canonical activation of both pathways. Thus, the modulation of alternative ways of their activation, such as the epigenetic regulation of Nrf2 and NF-κB by phytochemicals, should be more deeply investigate.

Currently, limited evidence exists demonstrating the modulation of Nrf2 and NF-κB in humans. Therefore, well-controlled human intervention trials are needed to confirm the findings from in vitro and animal studies.

Many phytochemicals demonstrate synergism in modulating Nrf2 or NF-κB pathways. Thus, their combinations, particularly that resembling a natural food matrix, may improve this effect. In contrast to the natural food matrix in phytochemical combinations, higher concentrations of these compounds may be applied, thus assuring the desired effect.

Therefore, the application of phytochemical combinations as modulators of Nrf2 and NF-κB and ultimately cancer prevention or therapy seems to be an attractive approach. However, several problems must be solved before the specific phytochemical combinations can be applied for this purpose.

In this regard the ratio of given compounds must be selected based on the detailed experimental data derived from different models.

Equally important is the evaluation of the type of interactions. Similarly, as in the case of single phytochemicals, their bioavailability in combinations must be assessed.

## Figures and Tables

**Table 1:** Modulation of Nrf2 and NF-kB pathways by selected phytochemicals in vitro and in vivo model.

Phytochemical	Source	Experimental Model	Concentrations	Effect on NF-ĸB	Effect on Nrf2	Ref.
**Xanthohumol**/Chalcone	*Humulus lupulus*	Human pancreatic cancer cells (PANC-1 cells)	* 5 µM and 10 µM		↑ expression of Nrf2, binding Nrf2 to DNA and expression of antioxidant enzymes (SOD, NQO1, GSTP)	[53]
		PANC-1 cells	5 µM and 10 µM	↓ expression of NF-ĸB, binding NF-ĸBp65 and NF-ĸBp50 to DNA, and expression of COX-2		[53]
**Apigenin**/Flavone	*Matricaria chamomilla*	Human hepatocellular liver carcinoma cells (HepG2 cells)	6.25 µM		↓ mRNA and protein levels of Nrf2, HO-1	[55]
		HepG2 cells	10 µg/mL	↓ mRNA and protein levels of NF-ĸBp50 and NF-ĸBp65		[56]
**Phenethyl isothiocyanate**/Isothiocyanate	*Brassicaceae*	PANC-1 cells	5 µM and 10 µM		↑ expression of Nrf2, binding Nrf2 to DNA, expression of SOD, NQO1, GSTP	[53]
		PANC-1 cells	5 µM and 10 µM	↓ expression of NF-ĸB, binding NF-ĸBp65 and NF-ĸBp50 to DNA, and expression of COX-2		[53]
**Sulforaphane**/Isothiocyanate	*Brassicaceae*	Human breast adenocarcinoma cells (MCF-7 cells, MDA-MB231 cells)	5 µM and 10 µM 20 µM		↑ expression of Nrf2, and GSTP, NQO1	[57]
		Murine macrophage (RAW264.7 cells)	5 µM and 15 µM	↓ translocation of NF-κB and expression of COX-2		[58]
**Sappanone**/Isoflavone	*Caesalpinia sappan*	RAW264.7 cells	30 µM		↑ nuclear translocation of Nrf2 and expression of HO-1, NQO1	[59]
		RAW264.7 cells	30 µM	↓ nuclear translocation of NF-ĸBp65		[59]
**Strigoterpenoid**/Terpenoid	*Pistachia terebinthus*	Human immortalized keratinocytes (HaCaT cells)	10 µM		↑ activation of Nrf2	[60]
		HaCaT cells	10 µM	↓ expression of NF-ĸB p65 ↑ expression of IKKβ		[60]
**Wogonin**/Flavone	*Scutelaria baicalensis*	Human erythroleukemic cells (K562 cells)	40 µM	↓ NF-ĸB activation and NF-ĸBp65 binding to DNA		[61]
		K562 cells	40 µM		↓ expression of Nrf2 and nuclear translocation of Nrf2	[61]
		MCF-7 cells	40 µM 60 µM		↓ expression of Nrf2 ↑ Keap1 protein level; ↓HO-1 and NQO1 protein level	[62]
**Curcumin**/diferuloylmethane	*Curcuma longa rhizomes*	MCF-7 cells	20 µM and 25 µM and 40 µM		↑ expression and protein of Nrf2	[63]
		Human lymphocytic leukemia cells (REH cells)	20 µM	↓expression of NF-ĸB		[64]
**Oridinin**/Diterpenoid	*Rabdosia rubescens*	Human osteosarcoma cells (MG-63 cells HOS cells)	15 µM	↓ NF-ĸB activation and NF-ĸB p65 binding to DNA	↓ nuclear translocation of Nrf2, expression of HO-1 and NQO1	[65]
		Male BALB/c nude mice	30 mg/kg intraperitoneally	↓ nuclear translocation of NF-ĸB	↓ nuclear translocation of Nrf2	[65]
**Resveratrol**/Stilbene	*Vitis vinifera*	HaCaT cells	60 µM		↑ nuclear level of Nrf2 and GSTP protein level	[66]
		HaCaT cells	60 µM	↓NF-ĸBp65, IĸB kinase		[67]
		Female ICR mice	0.25 µmol and 1 µmol topically applied	↓expression of NF-ĸBp65 and COX-2, and IKK activity		[68]
		Female ACI rats	50 mg as a subcutaneous pellet		↑ expression and protein levels of Nrf2 and HO-1, NQO1	[69]
**Genistein**/Isoflavone	*Genista tinctoria*	MDA-MB-231 cells	50 µM	↓ expression of NF-ĸBp65 and p50, COX-2		[70]
		Laying Hen model ovarian cancer	** 52.48 mg/hen and 106.26 mg/hen		↑ expression of Nrf2 and HO-1	[71]
		Laying Hen model ovarian cancer	52.48 mg/hen and 106.26 mg/hen	↓expression of NF-ĸB		[71]
**Epigallocatechin gallate**/Flavonoid	*Camellia sinensis*	Human prostate cancer cells (DU145 cells)	40 µg/mL	↓ nuclear translocation of NF-ĸBp65		[72]
		Sprague-Dawley rats	40 mg/kg intraperitoneally		↑ expression and protein level of Nrf2 and HO-1	[73]
**Arctigenin**/Lignan	*Fructus arctii*	SD male rats	20 mg/kg orally	↓ expression of NF-ĸB, COX-2		[74]
		SD male rats	20 mg/kg orally		↑ expression of SOD	[74]
**Piperine**/Alkaloid	*Piper nigrum*	Male Wistar rats	30 mg/kg and 60 mg/kg orally		↑ expression of Nrf2, HO-1, NOQ1	[75]
		Male Wistar rats	30 mg/kg and 60 mg/kg orally	↓ expression of NF-ĸB, iNOS, COX-2		[75]
**Quercetin**/Flavonoid	*Pinus banksiana*	Male ICR mice	40 mg/kg and 80 mg/kg gavage		↑ nuclear translocation of Nrf2 and expression of HO-1	[76]
		Male ICR mice	40 mg/kg and 80 mg/kg gavage	↓ nuclear translocation of NF-ĸBp65		[76]

* The concentrations of the phytochemicals in in vitro studies are quoted in µM or µg/mL; ** concentrations of phytochemicals in in vivo studies are quoted in mg/kg or mg/animal.

## Data Availability

Not applicable.

## References

[1] Albini A., Tosetti F., Li V.W., Noonan D.M., Li W.W. (2012). Cancer Prevention by Targeting Angiogenesis. Nat. Rev. Clin. Oncol..

[2] Hoesel B., Schmid J.A. (2013). The Complexity of NF-ΚB Signaling in Inflammation and Cancer. Mol. Cancer.

[3] Nicolas A., Carré M., Pasquier E. (2018). Metronomics: Intrinsic Anakoinosis Modulator?. Front. Pharmacol..

[4] Dinkova-Kostova A.T., Liby K.T., Stephenson K.K., Holtzclaw W.D., Gao X., Suh N., Williams C., Risingsong R., Honda T., Gribble G.W. (2005). Extremely Potent Triterpenoid Inducers of the Phase 2 Response: Correlations of Protection against Oxidant and Inflammatory Stress. Proc. Natl. Acad. Sci. USA.

[5] Singh S., Aggarwal B.B. (1995). Activation of Transcription Factor NF-ΚB Is Suppressed by Curcumin (Diferuloylmethane) (∗). J. Biol. Chem..

[6] Haque A., Brazeau D., Amin A.R. (2021). Perspectives on Natural Compounds in Chemoprevention and Treatment of Cancer: An Update with New Promising Compounds. Eur. J. Cancer.

[7] Jung B.-J., Yoo H.-S., Shin S., Park Y.-J., Jeon S.-M. (2018). Dysregulation of NRF2 in Cancer: From Molecular Mechanisms to Therapeutic Opportunities. Biomol. Ther..

[8] Saha S., Buttari B., Panieri E., Profumo E., Saso L. (2020). An Overview of Nrf2 Signaling Pathway and Its Role in Inflammation. Molecules.

[9] Krajka-Kuźniak V., Paluszczak J., Baer-Dubowska W. (2017). The Nrf2-ARE Signaling Pathway: An Update on Its Regulation and Possible Role in Cancer Prevention and Treatment. Pharmacol. Rep..

[10] Silva-Islas C.A., Maldonado P.D. (2018). Canonical and Non-Canonical Mechanisms of Nrf2 Activation. Pharmacol. Res..

[11] Shah S.Z.A., Zhao D., Hussain T., Sabir N., Mangi M.H., Yang L. (2018). P62-Keap1-NRF2-ARE Pathway: A Contentious Player for Selective Targeting of Autophagy, Oxidative Stress and Mitochondrial Dysfunction in Prion Diseases. Front. Mol. Neurosci..

[12] Sun X., Ou Z., Chen R., Niu X., Chen D., Kang R., Tang D. (2016). Activation of the P62-Keap1-NRF2 Pathway Protects against Ferroptosis in Hepatocellular Carcinoma Cells. Hepatology.

[13] Raghunath A., Sundarraj K., Arfuso F., Sethi G., Perumal E. (2018). Dysregulation of Nrf2 in Hepatocellular Carcinoma: Role in Cancer Progression and Chemoresistance. Cancers.

[14] Panieri E., Saso L. (2019). Potential Applications of NRF2 Inhibitors in Cancer Therapy. Oxid. Med. Cell Longev..

[15] Song M.-Y., Lee D.-Y., Chun K.-S., Kim E.-H. (2021). The Role of NRF2/KEAP1 Signaling Pathway in Cancer Metabolism. Int. J. Mol. Sci..

[16] Pires B.R.B., Silva R.C.M.C., Ferreira G.M., Abdelhay E. (2018). NF-KappaB: Two Sides of the Same Coin. Genes.

[17] Liu H., Xu H., Huang K. (2017). Selenium in the Prevention of Atherosclerosis and Its Underlying Mechanisms. Metallomics.

[18] Giridharan S., Srinivasan M. (2018). Mechanisms of NF-ΚB P65 and Strategies for Therapeutic Manipulation. J. Inflamm. Res..

[19] Yu H., Lin L., Zhang Z., Zhang H., Hu H. (2020). Targeting NF-ΚB Pathway for the Therapy of Diseases: Mechanism and Clinical Study. Signal Transduct. Target Ther..

[20] Wan F., Lenardo M.J. (2009). Specification of DNA Binding Activity of NF-KappaB Proteins. Cold Spring Harb. Perspect. Biol..

[21] Rius-Pérez S., Pérez S., Martí-Andrés P., Monsalve M., Sastre J. (2020). Nuclear Factor Kappa B Signaling Complexes in Acute Inflammation. Antioxid. Redox Signal..

[22] Verzella D., Pescatore A., Capece D., Vecchiotti D., Ursini M.V., Franzoso G., Alesse E., Zazzeroni F. (2020). Life, Death, and Autophagy in Cancer: NF-ΚB Turns up Everywhere. Cell Death Dis..

[23] Sandberg M., Patil J., D’Angelo B., Weber S.G., Mallard C. (2014). NRF2-Regulation in Brain Health and Disease: Implication of Cerebral Inflammation. Neuropharmacology.

[24] Wardyn J.D., Ponsford A.H., Sanderson C.M. (2015). Dissecting Molecular Cross-Talk between Nrf2 and NF-ΚB Response Pathways. Biochem. Soc. Trans.

[25] Liu G.-H., Qu J., Shen X. (2008). NF-ΚB/P65 Antagonizes Nrf2-ARE Pathway by Depriving CBP from Nrf2 and Facilitating Recruitment of HDAC3 to MafK. Biochim. Biophys. Acta Mol. Cell Res..

[26] Kaspar J.W., Niture S.K., Jaiswal A.K. (2009). Nrf2:INrf2 (Keap1) Signaling in Oxidative Stress. Free Radic. Biol. Med..

[27] González-Vallinas M., González-Castejón M., Rodríguez-Casado A., Ramírez de Molina A. (2013). Dietary Phytochemicals in Cancer Prevention and Therapy: A Complementary Approach with Promising Perspectives. Nutr. Rev..

[28] Rue E.A., Rush M.D., van Breemen R.B. (2018). Procyanidins: A Comprehensive Review Encompassing Structure Elucidation via Mass Spectrometry. Phytochem. Rev..

[29] Rasouli H., Farzaei M.H., Khodarahmi R. (2017). Polyphenols and Their Benefits: A Review. Int. J. Food Prop..

[30] Galluzzi L., Vitale I., Aaronson S.A., Abrams J.M., Adam D., Agostinis P., Alnemri E.S., Altucci L., Amelio I., Andrews D.W. (2018). Molecular Mechanisms of Cell Death: Recommendations of the Nomenclature Committee on Cell Death 2018. Cell Death Differ..

[31] Shin S.A., Joo B.J., Lee J.S., Ryu G., Han M., Kim W.Y., Park H.H., Lee J.H., Lee C.S. (2020). Phytochemicals as Anti-Inflammatory Agents in Animal Models of Prevalent Inflammatory Diseases. Molecules.

[32] Clifford T., Acton J.P., Cocksedge S.P., Davies K.A.B., Bailey S.J. (2021). The Effect of Dietary Phytochemicals on Nuclear Factor Erythroid 2-Related Factor 2 (Nrf2) Activation: A Systematic Review of Human Intervention Trials. Mol. Biol. Rep..

[33] Gugliandolo A., Bramanti P., Mazzon E. (2020). Activation of Nrf2 by Natural Bioactive Compounds: A Promising Approach for Stroke?. Int. J. Mol. Sci..

[34] Baer-Dubowska W., Szaefer H., Majchrzak-Celińska A., Krajka-Kuźniak V. (2020). Tannic Acid: Specific Form of Tannins in Cancer Chemoprevention and Therapy-Old and New Applications. Curr. Pharmacol. Rep..

[35] Clifford T., Howatson G., West D.J., Stevenson E.J. (2015). The Potential Benefits of Red Beetroot Supplementation in Health and Disease. Nutrients.

[36] Da Silva D.V.T., Baião D.D.S., Ferreira V.F., Paschoalin V.M.F. (2020). Betanin as a Multipath Oxidative Stress and Inflammation Modulator: A Beetroot Pigment with Protective Effects on Cardiovascular Disease Pathogenesis. Crit. Rev. Food Sci. Nutr..

[37] Krajka-Kuźniak V., Paluszczak J., Szaefer H., Baer-Dubowska W. (2013). Betanin, a Beetroot Component, Induces Nuclear Factor Erythroid-2-Related Factor 2-Mediated Expression of Detoxifying/Antioxidant Enzymes in Human Liver Cell Lines. Br. J. Nutr..

[38] Harish V., Haque E., Śmiech M., Taniguchi H., Jamieson S., Tewari D., Bishayee A. (2021). Xanthohumol for Human Malignancies: Chemistry, Pharmacokinetics and Molecular Targets. Int. J. Mol. Sci..

[39] Krajka-Kuźniak V., Paluszczak J., Baer-Dubowska W. (2013). Xanthohumol Induces Phase II Enzymes via Nrf2 in Human Hepatocytes in Vitro. Toxicol. Vitro.

[40] Lee I.-S., Lim J., Gal J., Kang J.C., Kim H.J., Kang B.Y., Choi H.J. (2011). Anti-Inflammatory Activity of Xanthohumol Involves Heme Oxygenase-1 Induction via NRF2-ARE Signaling in Microglial BV2 Cells. Neurochem. Int..

[41] Lv H., Liu Q., Wen Z., Feng H., Deng X., Ci X. (2017). Xanthohumol Ameliorates Lipopolysaccharide (LPS)-Induced Acute Lung Injury via Induction of AMPK/GSK3β-Nrf2 Signal Axis. Redox Biol..

[42] Lněničková K., Šadibolová M., Matoušková P., Szotáková B., Skálová L., Boušová I. (2020). The Modulation of Phase II Drug-Metabolizing Enzymes in Proliferating and Differentiated CaCo-2 Cells by Hop-Derived Prenylflavonoids. Nutrients.

[43] Kim H., Ramirez C.N., Su Z.-Y., Kong A.-N.T. (2016). Epigenetic Modifications of Triterpenoid Ursolic Acid in Activating Nrf2 and Blocking Cellular Transformation of Mouse Epidermal Cells. J. Nutr. Biochem..

[44] Chian S., Thapa R., Chi Z., Wang X.J., Tang X. (2014). Luteolin Inhibits the Nrf2 Signaling Pathway and Tumor Growth in Vivo. Biochem. Biophys. Res. Commun..

[45] Arlt A., Sebens S., Geismann C., Grossmann M., Krise M.L., Schreiber S., Schäfer H. (2013). Inhibition of the Nrf2 Transcription Factor by the Alkaloid Through Decreased Proteosomal Gene Expression and Proteosome Activity. Oncogene.

[46] Ohnuma T., Matsumoto T., Itoi A., Kawana A., Nishiyama T., Ogura K., Hiratsuka A. (2011). Enhanced Sensitivity of A549 Cells to the Cytotoxic Action of Anticancer Drugs via Suppression of Nrf2 by Procyanidins from Cinnamomi Cortex Extract. Biochem. Biophys. Res. Commun..

[47] De Prax M.C.A., Ferro K.P.V., Santos I., Torello C.O., Salazar-Terreros M., Olalla Saad S.T. (2019). NRF2 Is Targeted By the Polyphenol Quercetin and Induces Apoptosis, in Part, through up Regulation of Pro Apoptotic Mirs. Blood.

[48] Bhattacharjee S., Dashwood R.H. (2020). Epigenetic Regulation of NRF2/KEAP1 by Phytochemicals. Antioxidants.

[49] Pulido-Moran M., Moreno-Fernandez J., Ramirez-Tortosa C., Ramirez-Tortosa M. (2016). Curcumin and Health. Molecules.

[50] Nelson K.M., Dahlin J.L., Bisson J., Graham J., Pauli G.F., Walters M.A. (2017). The Essential Medicinal Chemistry of Curcumin. J. Med. Chem..

[51] Cichocki M., Blumczyńska J., Baer-Dubowska W. (2010). Naturally Occurring Phenolic Acids Inhibit 12-O-Tetradecanoylphorbol-13-Acetate Induced NF-ΚB, INOS and COX-2 Activation in Mouse Epidermis. Toxicology.

[52] Reddy M.K., Alexander-Lindo R.L., Nair M.G. (2005). Relative Inhibition of Lipid Peroxidation, Cyclooxygenase Enzymes, and Human Tumor Cell Proliferation by Natural Food Colors. J. Agric. Food Chem..

[53] Krajka-Kuźniak V., Cykowiak M., Szaefer H., Kleszcz R., Baer-Dubowska W. (2020). Combination of Xanthohumol and Phenethyl Isothiocyanate Inhibits NF-ΚB and Activates Nrf2 in Pancreatic Cancer Cells. Toxicol. Vitro.

[54] Guo W., Xu B., Zheng B., Du J., Liu S. (2020). The Analysis of the Anti-Tumor Mechanism of Ursolic Acid Using Connectively Map Approach in Breast Cancer Cells Line MCF-7. Cancer Manag. Res..

[55] Paredes-Gonzalez X., Fuentes F., Jeffery S., Saw C.L.L., Shu L., Su Z.Y., Kong A.N.T. (2015). Induction of Nrf2-Mediated Gene Expression by Dietary Phytochemical Flavones Apigenin and Luteolin. Biopharm. Drug Dispos..

[56] Witkowska-Banaszczak E., Krajka-Kuźniak V., Papierska K. (2020). The Effect of Luteolin 7-Glucoside, Apigenin 7-Glucoside and Succisa Pratensis Extracts on NF-ΚB Activation and α-Amylase Activity in HepG2 Cells. Acta Biochim. Pol..

[57] Licznerska B., Szaefer H., Krajka-Kuźniak V. (2021). R-Sulforaphane Modulates the Expression Profile of AhR, ERα, Nrf2, NQO1, and GSTP in Human Breast Cell Lines. Mol. Cell Biochem..

[58] Heiss E., Herhaus C., Klimo K., Bartsch H., Gerhäuser C. (2001). Nuclear Factor Kappa B Is a Molecular Target for Sulforaphane-Mediated Anti-Inflammatory Mechanisms. J. Biol. Chem..

[59] Lee S., Choi S.Y., Choo Y.Y., Kim O., Tran P.T., Dao C.T., Min B.S., Lee J.H. (2015). Sappanone A Exhibits Anti-Inflammatory Effects via Modulation of Nrf2 and NF-κB. Int. Immunopharmacol..

[60] Rogati F., Millán E., Appendino G., Correa A., Caprioglio D., Minassi A., Muňoz E. (2019). Identification of a Strigoterpenoid with Dual Nrf2 and NF-ĸB Modulatory Activity. ACS Med. Chem. Lett..

[61] Xu X., Zhang X., Zhang Y., Yang L., Liu Y., Huang S., Lu L., Kong L., Li Z., Guo Q. (2017). Wogonin Reserved Resistant Human Myelogenous Leukemia Cells via Inhibiting Nrf2 Signaling by Stat3/NF-ĸB Inactivation. Sci. Rep..

[62] Zhong Y., Zhang F., Sun Z., Zhou W., Li Z.Y., You Q.D., Guo Q.L., Hu R. (2013). Drug Resistance Associates with Activation of Nrf2 in MCF-7/DOX Cells, and Wogonin Reserves it By Down-Regulating Nrf2-Mediated Cellular Defense Response. Mol. Carcinog..

[63] Chen B., Zhang Y., Wang Y., Rao J., Jiang X., Xu Z. (2014). Curcumin Inhibits Proliferation of Breast Cancer Cells Through Nrf2-Mediated Down-Regulation of Fen1 Expression. J. Steroid Biochem. Mol. Biol..

[64] Pimentel-Gutiérrez H.J., Bobadilla-Morales L., Barba-Barba C.C., Ortega-De-La-Torre C., Sánchez-Zubieta F.A., Corona-Rivera J.R., González-Quezada B.A., Armendáriz-Borunda J.S., Silva-Cruz R., Corona-Rivera A. (2016). Curcumin Potentiates the Effect of Chemotherapy Against Acute Lymphoblastic Leukemia Cells Via Downregulation of NF-ĸB. Oncol. Lett..

[65] Lu Y., Sun Y., Zhu J., Yu L., Jiang X., Zhang J., Dong X., Ma B., Zhang Q. (2018). Oridonin Exerts Anticancer Effect on Osteosarcoma by Activating PPAR-γ and Inhibiting Nrf2 Pathway. Cell Death Dis..

[66] Krajka-Kuźniak V., Szaefer H., Stefański T., Sobiak S., Cichocki M., Baer-Dubowska W. (2014). The Effect of Resveratrol and Its Methylthioderivatives on the Nrf2-ARE Pathway in Mouse Epidermis and HaCaT Keratinocytes. Cell Mol. Biol. Lett..

[67] Szaefer H., Cichocki M., Krajka-Kuźniak V., Stefański T., Sobiak S., Licznerska B., Baer-Dubowska W. (2014). The Effect of Resveratrol and Its Methylthioderivatives on NF-ĸB and AP-1 signaling Pathways in HaCaT Keratinocytes. Pharmacol. Rep..

[68] Kundu J.K., Shin Y.K., Kim S.H., Surh Y.-J. (2006). Resveratrol Inhibits Phorbol Ester-Induced Expression of COX-2 and Activation of NF-KappaB in Mouse Skin by Blocking IkappaB Kinase Activity. Carcinogenesis.

[69] Singh B., Shoulson R., Chatterjee A., Ronghe A., Bhat N.K., Dim D.C., Bhat H.K. (2014). Resveratrol Inhibits Estrogen-Induced Breast Carcinogenesis Through Induction of Nrf2-Mediated Protective Pathways. Carcinogenesis.

[70] Gong L., Li Y., Nedeljkovic-Kurepa A., Sarkar F.H. (2003). Inactivation of NF-KappaB by Genistein Is Mediated via Akt Signaling Pathway in Breast Cancer Cells. Oncogene.

[71] Sahin K., Yenice E., Bilir B., Orhan C., Tuzcu M., Sahin N., Ozercan I.H., Kabil N., Ozpolat B., Kucuk O. (2019). Genistein Prevents Development of Spontaneous Ovarian Cancer and Inhibits Tumor Growth in Hen Model. Cancer Prev. Res..

[72] Mukherjee S., Siddiqui M.A., Dayal S., Ayoub Y.Z., Malathi K. (2014). Epigallocatechin-3-gallate Suppresses Proinflammatory Cytokines and Chemokines Induced by Toll-like Receptor 9 Agonists in Prostate Cancer Cells. J. Inflamm. Res..

[73] Han J., Wang M., Jing X., Shi H., Ren M., Lou H. (2014). (-)-Epigallocatechin Gallate Protects against Cerebral Ischemia-Induced Oxidative Stress via Nrf2/ARE Signaling. Neurochem Res..

[74] Zhang N., Dou D., Ran X., Kang T. (2018). Neuroprotective Effect of Arctigenin against Neuroinflammation and Oxidative Stress Induced by Rotenone. RSC Adv..

[75] Rehman M.U., Rashid S., Arafah A., Qamar W., Alsaffar R.M., Ahmad A., Almatroudi N.M., Alqahtani S.M.A., Rashid S.M., Ahmad S.B. (2020). Piperine Regulates Nrf-2/Keap-1 Signalling and Exhibits Anticancer Effect in Experimental Colon Carcinogenesis in Wistar Rats. Biology.

[76] Liu C.-M., Ma J.-Q., Xie W.-R., Liu S.-S., Feng Z.-J., Zheng G.-H., Wang A.-M. (2015). Quercetin Protects Mouse Liver against Nickel-Induced DNA Methylation and Inflammation Associated with the Nrf2/HO-1 and P38/STAT1/NF-ΚB Pathway. Food Chem. Toxicol..

[77] Szaefer H., Krajka-Kuźniak V., Ignatowicz E., Adamska T., Baer-Dubowska W. (2014). Evaluation of the Effect of Beetroot Juice on DMBA-Induced Damage in Liver and Mammary Gland of Female Sprague-Dawley Rats. Phytother. Res..

[78] Tan M.L., Hamid S.B.S. (2021). Beetroot as a Potential Functional Food for Cancer Chemoprevention, a Narrative Review. J. Cancer Prev..

[79] Krajka-Kuźniak V., Szaefer H., Ignatowicz E., Adamska T., Baer-Dubowska W. (2012). Beetroot Juice Protects against N-Nitrosodiethylamine-Induced Liver Injury in Rats. Food Chem. Toxicol..

[80] El Gamal A.A., Al Said M.S., Raish M., Al-Sohaibani M., Al-Massarani S.M., Ahmad A., Hefnawy M., Al-Yahya M., Basoudan O.A., Rafatullah S. (2014). Beetroot (*Beta vulgaris* L.) Extract Ameliorates Gentamicin-Induced Nephrotoxicity Associated Oxidative Stress, Iflammation, and Apoptosis in Rodent Model. Mediat. Inflamm..

[81] Hyun T.K., Jang K.-I. (2016). Apple as a Source of Dietary Phytonutrients: An Update on the Potential Health Benefits of Apple. EXCLI J..

[82] Lodi A., Saha A., Lu X., Wang B., Sentandreu E., Collins M., Kolonin M.G., DiGiovanni J., Tiziani S. (2017). Combinatorial Treatment with Natural Compounds in Prostate Cancer Inhibits Prostate Tumor Growth and Leads to Key Modulations of Cancer Cell Metabolism. NPJ Precis. Onc..

[83] Tremmel L., Rho O., Slaga T.J., DiGiovanni J. (2019). Inhibition of Skin Tumor Promotion by TPA Using a Combination of Topically Applied Ursolic Acid and Curcumin. Mol. Carcinog..

[84] Saw C.L.L., Guo Y., Yang A.Y., Paredes-Gonzalez X., Ramirez C., Pung D., Kong A.-N.T. (2014). The Berry Constituents Quercetin, Kaempferol, and Pterostilbene Synergistically Attenuate Reactive Oxygen Species: Involvement of the Nrf2-ARE Signaling Pathway. Food Chem Toxicol..

[85] Hackman G.L., Collins M., Lu X., Lodi A., DiGiovanni J., Tiziani S. (2020). Predicting and Quantifying Antagonistic Effects of Natural Compounds Given with Chemotherapeutic Agents: Applications for High-Throughput Screening. Cancers.

[86] Probst B.L., McCauley L., Trevino I., Wigley W.C., Ferguson D.A. (2015). Cancer Cell Growth Is Differentially Affected by Constitutive Activation of NRF2 by KEAP1 Deletion and Pharmacological Activation of NRF2 by the Synthetic Triterpenoid, RTA 405. PLoS ONE.

[87] Cykowiak M., Krajka-Kuźniak V., Baer-Dubowska W. (2021). Combinations of Phytochemicals More Efficiently than Single Components Activate Nrf2 and Induce the Expression of Antioxidant Enzymes in Pancreatic Cancer Cells. Nutr. Cancer.

[88] Wang P., Wang B., Chung S., Wu Y., Henning S.M., Vadgama J.V. (2014). Increased Chemopreventive Effect by Combining Arctigenin, Green Tea Polyphenol and Curcumin in Prostate and Breast Cancer Cells. RSC Adv..

[89] Saw C.L.L., Cintron M., Wu T.Y., Guo Y., Huang Y., Jeong W.S., Kong A.N.T. (2011). Pharmacodynamics of dietary phytochemical indoles I3C and DIM: Induction of Nrf2-mediated Phase II Drug Metabolizing and Antioxidant Genes and Synergism with isothiocyanates. Biopharm. Drug Dispos..

[90] Cheung K.L., Khor T.O., Kong A.-N. (2009). Synergistic Effect of Combination of Phenethyl Isothiocyanate and Sulforaphane or Curcumin and Sulforaphane in the Inhibition of Inflammation. Pharm. Res..

[91] Masuelli L., Di Stefano E., Fantini M., Mattera R., Benvenuto M., Marzocchella L., Sacchetti P., Focaccetti C., Bernardini R., Tresoldi I. (2014). Resveratrol Potentiates the in Vitro and in Vivo Anti-Tumoral Effects of Curcumin in Head and Neck Carcinomas. Oncotarget.

[92] Majumdar A.P.N., Banerjee S., Nautiyal J., Patel B.B., Patel V., Du J., Yu Y., Elliott A.A., Levi E., Sarkar F.H. (2009). Curcumin Synergizes with Resveratrol to Inhibit Colon Cancer. Nutr. Cancer.

[93] Guimaraes-Stabili M.R., de Aquino S.G., de Almeida Curylofo F., Tasso C.O., Rocha F.R.G., de Medeiros M.C., de Pizzol J.P., Cerri P.S., Romito G.A., Rossa C. (2019). Systemic Administration of Curcumin or Piperine Enhances the Periodontal Repair: A Preliminary Study in Rats. Clin Oral. Investig..

